# Molecular Markers of Therapy-Resistant Glioblastoma and Potential Strategy to Combat Resistance

**DOI:** 10.3390/ijms19061765

**Published:** 2018-06-14

**Authors:** Ha S. Nguyen, Saman Shabani, Ahmed J. Awad, Mayank Kaushal, Ninh Doan

**Affiliations:** 1 Department of Neurosurgery, Medical College of Wisconsin, Milwaukee, WI 53226, USA; hsnguyen@mcw.edu (H.S.N.); sshabani@mcw.edu (S.S.); aawad@mcw.edu (A.J.A.); mkaushal@mcw.edu (M.K.); 2 Faculty of Neurosurgery, California Institute of Neuroscience, Thousand Oaks, CA 91360, USA; 3 Faculty of Medicine and Health Sciences, An-Najah National University, Nablus 11941, Palestine; 4 Department of Neurosurgery, Mitchell Cancer Institute, University of South Alabama, Mobile, AL 36688, USA

**Keywords:** glioblastoma, acid ceramidase, acid ceramidase inhibitors, carmofur, radioresistance, radiation, sphingosine, sphingosine-1-phosphate, S1P

## Abstract

Glioblastoma (GBM) is the most common primary malignant tumor of the central nervous system. With its overall dismal prognosis (the median survival is 14 months), GBMs demonstrate a resounding resilience against all current treatment modalities. The absence of a major progress in the treatment of GBM maybe a result of our poor understanding of both GBM tumor biology and the mechanisms underlying the acquirement of treatment resistance in recurrent GBMs. A comprehensive understanding of these markers is mandatory for the development of treatments against therapy-resistant GBMs. This review also provides an overview of a novel marker called acid ceramidase and its implication in the development of radioresistant GBMs. Multiple signaling pathways were found altered in radioresistant GBMs. Given these global alterations of multiple signaling pathways found in radioresistant GBMs, an effective treatment for radioresistant GBMs may require a cocktail containing multiple agents targeting multiple cancer-inducing pathways in order to have a chance to make a substantial impact on improving the overall GBM survival.

## 1. Introduction

Glioblastoma (GBM) is the most common primary malignant tumor of the central nervous system. With its overall dismal prognosis, GBMs demonstrate a resounding resilience against all current treatment modalities. The estimated overall survival of GBM patients is less than 1.5 years, and the 5-year survival rate is 5% [1,2,3]. The median age of diagnosis of GBM has increased to 64 years over the last decades, and the top incidence is 15.24/100,000 populations diagnosed within the age range of 75–84 years [1,2,3]. While radiation is the only proved cause of GBM, only a minority of patients develop GBMs following exposure to radiation [4]. The etiology of GBM remains to be discovered, and fewer than 5% of patients have a germline mutation which increases the risk for developing GBMs [5,6]. Symptoms at presentation are based on the location of GBMs. Eloquent-area tumors often engender symptoms ranging from numbness, weakness, and visual disturbance to language deficits, while tumors in other areas (including the non-dominant frontal and temporal lobes or the corpus callosum) may induce non-specific symptoms (such as seizures, which can be controlled with anticonvulsant medications in 25% of patients with newly diagnosed GBMs [7]). However, new emerging data have suggested that the administration of anticonvulsants may not be beneficial and can produce significant, undesired effects in GBM patients without seizures [8,9]. The presenting symptoms include headaches (~60%), memory loss (~40%), and cognitive, language, or motor deficits (~40%) [10]. The most common imaging modality to diagnose GBMs is magnetic resonance imaging (MRI) of the brain with and without gadolinium contrast. A heterogeneous ring-enhancement with area of central necrosis is the signature feature of GBMs; infrequently, GBMs can be multi-focal. Headache has been attributed to peritumoral edema, which can cause a major midline shift or mass effect [11]. Steroids such as dexamethasone are commonly employed to provide relief from headache or deficits by reducing the peritumoral edema, generally within 48 h [12,13]. Another therapy aimed at reducing the peritumoral edema is based on the anti-angiogenesis antibody bevacizumab, but it has been shown not to affect the overall survival in patients with newly diagnosed GBMs [14,15]. GBMs having certain prognostic biomarker mutations, such as isocitrate dehydrogenase (IDH), may present, on MRI, with characteristic features, such as a large non-enhancing mass with pial invasion, decreased blood flow, minimal edema and necrosis, and a tendency for the frontal and temporal lobes [16,17]. Following surgery, resected GBM tissues are formalin-fixed and paraffin-embedded prior to undergoing histopathology examinations, which characteristically show palisading necrosis, marked pleomorphism, a high mitotic index, and microvascular proliferation. Additionally, these GBM tissues are also further examined by immunostaining or sequencing for IDH mutations, O6-methylguanine methyltransferase (MGMT) methylation, and other prognostic biomarkers, which will be discussed in detail below [18,19].

The absence of a major progress in the treatment of GBM may be a result of our poor understanding of both GBM tumor biology and the mechanisms underlying the acquirement of treatment resistance in recurrent GBMs. Others have proposed that glioblastoma stem-like cells (GSCs), carrying the cell membrane marker CD133, may play a significant role in the resistance of this cancer to chemotherapy and radiotherapy [20,21,22,23]. The higher expression levels of CD133 have been linked to poorer prognosis [23]. Proteins or signaling pathways that maintain stemness may contribute to the development of therapy-resistant GBMs [24]. Novel druggable targets that have been reported to combat therapy-resistant GBMs include sodium pump α1 subunit, wingless-type MMTV integration site family member (*Wnt*)/β-catenin, sonic hedgehog/Glioma-associated oncogene (*SHH*/*GLI*), oligodendrocyte transcription factor 2(*OLIG2*), polycomb group RING finger protein 4 (*BMI1*), *NANOG*, and inhibitor of differentiation/DNA binding (*ID1*) [24,25]. More recently, circular RNAs (circRNAs) such as circSMARCA5, whose expression is downregulated in GBM samples as compared to control tissues, has been described to function as a novel tumor-suppressor, regulating the migration of GBM cells by modulating the oncoprotein that modulates cell migration, called RNA binding protein serine- and arginine-rich splicing factor 1 (SRSF1) [26]. 

A comprehensive understanding of established prognostic markers is mandatory for the development of treatments against therapy-resistant GBMs. In addition to discuss the established prognostic markers, this review also provides an overview of a novel marker called acid ceramidase (*ASAH1*) and its implications in the development of radioresistant GBMs. Multiple signaling pathways were found altered in radioresistant GBMs. Given the global alterations of multiple signaling pathways found in radioresistant GBMs, an effective treatment targeting radioresistant GBMs may require a cocktail containing multiple agents targeting multiple cancer-inducing pathways in order to have a chance to make a substantial impact on improving overall GBM survival. 

## 2. O6-Methylguanine Methyltransferase (*MGMT*)

**Figure 1:**
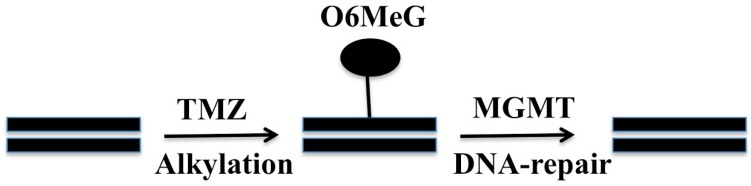
A cartoon showing temozolomide (TMZ) alkylating DNA at the O-6 position of guanine residues and the removal of this alkyl group by O6-methylguanine methyltransferase (MGMT) through a DNA repair process.

Alkylating agents, such as temozolomide (TMZ), attach an alkyl group to the DNA, frequently at the N-7 or O-6 positions of guanine residues (Figure 1) [27]. This process damages the DNA and triggers cell cycle death, unless the DNA is promptly repaired. O6-methylguanine methyltransferase (*MGMT*), a DNA repair protein, can hydrolyze the alkyl groups off guanine and impede the effectiveness of such chemotherapeutic agents [28]. Methylation of the *MGMT* promoter at CpG sites can suppress the gene and promote sensitivity to alkylating agents. Overall, up to 45–47% of GBMs exhibited methylation in prior studies [28,29]. The MGMT methylation status of tumors significantly correlates with progression-free survival (PFS) and overall survival (OS) for patients undergoing treatment with alkylating agents [28]. For newly diagnosed GBM, alkylating agents are a mainstay option irrespective of the MGMT methylation status [30]; for elderly patients, those with MGMT methylation may have a greater benefit from TMZ monotherapy than radiotherapy [31], while those without methylation may not benefit from alklylating agents. At recurrence, alterations in MGMT methylation status have not been detected [32]; moreover, the relationship with MGMT methylation persists at recurrence, and TMZ re-challenge is a sensible choice for patients with MGMT-methylated GBM [33]. 

Presently, molecular testing occurs via either quantitative methylation-specific PCR or pyrosequencing [34]. The former employs methylation-specific primer pairs to probe CpG islands with high methylation density; the latter enumerates the methylation sites at individual CpG sites through the “sequencing-by-synthesis” principle when nucleotides get incorporated by DNA polymerase [35]. To complicate matters, there is no accepted threshold for the number of methylated sites for a tumor to be classified as “methylated”; various detection methods yield methylation rates varying from 33% to 60% for the same group of GBM patients [36]. Moreover, additional research is required to elucidate the impact of the extent of methylation or the patterns of methylation on GBM survival [35]. 

## 3. Epidermal Growth Factor Receptor (EGFR)

Epidermal growth factor receptor (EGFR) is a transmembrane tyrosine kinase that functions as a critical player in pathways linked to cell proliferation, migration, and survival. EGFR activity can be augmented via gene amplification or EGFR variant III deletion mutation (EGFRvIII); the latter results in a truncated receptor that is constitutively active, promoting mitogenic cascades. EGFR amplification occurs in roughly 40–60% of GBM; EGFRvIII, which only occurs in a subset of those GBMs with EGFR amplification, arises in approximately 20–30% of GBM overall [35,37,38,39,40]. EGFR amplification is assessed via fluorescence in situ hybridization (FISH); EGFRvIII expression can be established by immunohistochemistry (IHC) [38]. 

Studies regarding the implications of EGFR amplification and EGFRvIII mutation have reported mixed, conflicting results regarding GBM survival [27,37,39]. Given the positive results in other types of cancers, researchers believed that receptor tyrosine kinase inhibitors could play a role in the treatment of GBM [39]. However, clinical trials for GBM designed to target EGFR have been disappointing [39]. The dearth of clinical effectiveness may be due to the inability of the examined drugs to cross the blood-brain barrier and/or the development of resistance through gained mutations [40]. 

## 4. Isocitrate Dehydrogenase (*IDH*)1/2

Isocitrate dehydrogenase (*IDH*) is a component of the Krebs cycle that converts isocitrate and cofactor NAD+ to carbon dioxide, NADH, and α-ketoglutarate. Since the initial discovery of IDH mutations in GBM [41], several studies have observed that *IDH* mutations occurred in approximately 8–13% of all GBMs, including greater than 80% of secondary GBM [42]. The most common mutations are IDH1 R132 and IDH2 R172, comprising roughly 90% of *IDH* mutations; the former is noted in more than 70% of grade 2/3 gliomas and in GBMs that progressed from these lower-grade tumors [41,43]. The mutations cause a buildup of the onco-metabolite d-2-hydroxy-glutarate, which can disturb DNA methylation, gene transcription, and histone alterations; moreover, mutations may decrease NAPDH formation, promoting oxidative stress and leading to DNA damage [27,35]. 

Several studies have documented survival benefits (OS and PFS) in gliomas with *IDH* mutations, ranging from an average of 12 to 30 months [27,42,43,44]. In addition, IDH mutations convey a higher sensitivity to TMZ and radiotherapy [45,46,47]. *IDH* mutations have also been correlated with improved MRI-defined enhancing disease, allowing larger resections [48]. At present, *IDH* mutations can be detected via sequencing or IHC [38]. Preclinical studies have demonstrated that small molecule inhibitors of mutant IDH can lower the intracellular levels of d-2-hydroxy-glutarate, overturn epigenetic dysregulation, and promote cellular differentiation [49].

## 5. 1p19q Co-Deletion

The unbalanced whole-arm translocation of the centromeric portions between chromosomes 1q and 19q is defined as 1p19q co-deletion. The recent WHO 2016 criteria utilize this co-deletion, along with an IDH mutation, to classify gliomas into the oligodendroglial phenotype. The co-deletion occurs in roughly 60–80% of grade 2 or 3 oligodendrogliomas, 20–50% of grade 2 or 3 oligoastrocytomas, and less than 10% of diffuse gliomas (together with GBM) [50]. For oligodendroglioma, this co-deletion has been associated with favorable survival as well as responsiveness to chemotherapy (PCV and temozolomide) and radiotherapy [51,52,53,54]. The reasoning behind this sensitivity to treatment remains elusive. Studies concerning the co-deletion in GB have reported mixed results [55,56]; however, a meta-analysis by Zhao et al. [57], incorporating 3408 gliomas across 28 studies, noted that 1p/19q co-deletion was associated with improved survival (PFS and OS) irrespective of the histological grade. Frequently, detection of the 1p19q co-deletion is completed through FISH; other methods include microsatellite analysis, PCR, and array comparative genomic hybridization [58]. 

## 6. α-Thalassemia/Mental Retardation Syndrome X-Linked (ATRX)

The *ATRX* (α-thalassemia/mental retardation syndrome X-linked) gene encodes a protein involved in genomic stability, chromatin remodeling, and DNA methylation [59]. Inactivation of the gene is highly linked to the ALT (alternative lengthening of telomeres) phenotype. ALT is a mechanism for the regulation of telomere length that is vital to cell survival and proliferation [59]. Its role in glioma biology has only recently been explored. *ATRX* mutation is frequently associated with IDH mutations, but rarely with 1p19q co-deletions [59]. For anaplastic gliomas, *ATRX* loss defines a subset of *IDH* mutants with a significantly longer median time to treatment failure (close to 24 months) [60]. By using a mouse model of *ATRX*-deficient GBM, Koschmann et al. suggested that *ATRX* mutations lead to a genetically erratic tumor. With no treatment, the tumor behaved rather aggressively; on the contrary, with treatment directed at double-stranded DNA damage, the overall survival improved [61]. Commonly, detection of *ATRX* loss is performed via IHC; other methods include PCR, sequencing, and Western blotting [35,38]. 

## 7. Telomerase Reverse Transcriptase (*TERT*)

Telomeres are nucleoprotein complexes (comprised of hundreds of repetitive nucleotide sequences) that bind the extremes of chromosomes to ensure chromosomal integrity [62]. Each cell division prompts telomere truncation until its depletion, which provokes cell dormancy or death [62]. Telomerase reverse transcriptase (*TERT*) is a subunit of telomerase, an enzyme that inserts additional nucleotides to telomeres [62]. For normal adult cells, telomerase is typically inactive [62]. Activating mutations in the *TERT* promoter are frequently reported in grade IV astrocytomas (up to 85% of GBM) and grade 2/3 oligodendrogliomas (close to 80%) [62,63,64]. TERT mutations are strongly correlated with 1p19q co-deletion, but not with either *IDH* mutations or *ATRX* loss [64]. Comparisons of groups based on the statuses of IDH, 1p19q, and TERT revealed that *TERT* mutation bestows better outcomes in gliomas with *TERT* mutant/*IDH* mutation/1p19q co-deletion, but poorer survival in GBM with *TERT* mutant/*IDH* mutation without 1p19q co-deletion [63]. Currently, detection of *TERT* mutations is completed via methyl-specific PCR; in addition, rapid intraoperative testing has been reported [65]. 

## 8. Acid Ceramidase (*ASAH1*) as a Druggable Target to Combat Multiple Therapy-Resistant Cancers

**Figure 2:**
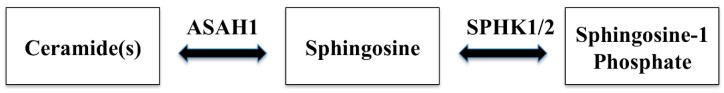
A schematic diagram of the sphingolipid signaling pathway, demonstrating the conversion of ceramides into sphingosine by acid ceramidase (*ASAH1*) and the subsequent transformation of sphingosine into sphingosine-1-phosphate by sphingosine kinase 1 or 2 (*SPHK1*, *SPHK2*).

*ASAH1*, initially discovered in rat brain homogenates and further characterized and purified from human urine in 1995, is a lysosomal cysteine amidase that catalyzes the transformation of ceramide into sphingosine and free fatty acid (Figure 2) [66,67,68,69,70,71,72]. Following this, sphingosine kinase 1 (*SPHK1*) or 2 (*SPHK2*) phosphorylates sphingosine to produce sphingosine-1-phosphate (S1P), which promotes GBM invasiveness via the upregulation of the urokinase plasminogen activator, its receptor, and the pro-invasive molecule *CCN1* (cysteine-rich angiogenic protein 61) (Figure 2) [69,72,73,74]. On the other hand, high levels of ceramides, carrying fatty acid side chains ranging from 14 to 26 carbons and generated via the action of ceramide synthases (*CerS*), promote apoptosis in cells that have undergone radio- and chemotherapy via the release of cytochrome c, leading to the activation of caspase-9 and caspase-3 [69,70,71,75,76,77,78,79]. Since its products are involved in the regulation of cell proliferation, multiple studies have linked *ASAH1* to multiple cancers such as melanoma, acute myeloid leukemia (AML), and colon and prostate cancers [80,81,82,83,84]. *ASAH1* has been proposed as an emerging drug target in AML [85]. Interestingly, over-expression of *ASAH1* in prostate cancer promotes resistance to chemotherapy. Prostate cancer upregulates *ASAH1* following radiation, which was described as a mechanism enabling the cancer to survive radiation [86]. Consequently, when the activity of *ASAH1* is suppressed with an *ASAH1* inhibitor named B13, the cells become more sensitive to chemotherapy and radiation as a result of the accumulation of intracellular ceramide up to cytotoxic levels, inducing apoptosis [81,87,88]. Similarly, the acid ceramidase inhibitor ceranib-2 also has activity against the growth of the breast cancer cell lines MCF-7 and MDA MB-231 via the activation of stress-activated protein kinase/c-Jun N-terminal kinase and p38 mitogen-activated protein kinase apoptotic pathways and the inhibition of the Akt pathway [89]. The *Wnt*/*β-catenin* signaling pathway appears to play a role in suppressing the proliferation and metastatic potential of cervical cancers when these tumors were treated with a recently identified *ASAH1* inhibitor called carmofur [90,91]. The clinical application of carmofur has been attempted and it offered benefits when carmofur was used an adjuvant in patients with early breast cancer in a postoperative setting [92]. 

## 9. ASAH1-Induced Radioresistance in GBM

**Figure 3:**
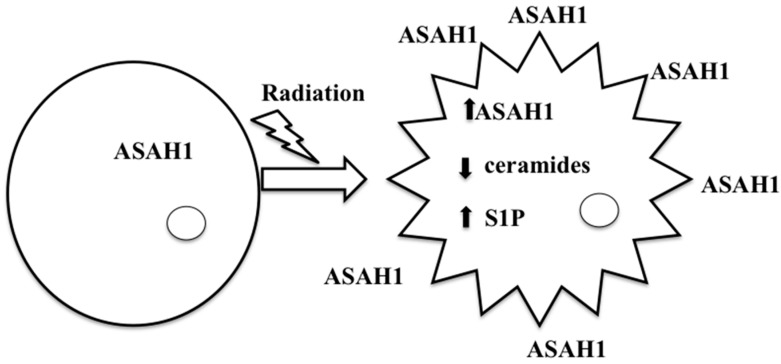
Schematic diagram is shown describing the molecular changes occurring in a glioblastoma (GBM) cell following radiation: increased secretion of *ASAH1*, increased intracellular levels of ASAH1 and S1P and decreased level of ceramides. ↑: Upregulation, ↓: Downregulation

The sphingolipid pathway was initially implicated in GBM in several studies, by showing that S1P augments the migratory response of the GBM cell line U87MG and that S1P level is significantly higher in GBM tissues compared to the normal gray matter [93,94]. We provided further evidence of the important role that the sphingolipid pathway plays in GBM. We showed that ASAH1 level was negatively correlated with GBM survival [95]. To study the role ASAH1 plays in radioresistant GBM, we developed a stable radioresistant GBM model, in which U87 GBM cells were irradiated, and the surviving cells were perpetuated [96]. In this model, we demonstrated that intracellular ASAH1 was upregulated, and its secretion into extracellular space was also increased in the adult GBM cell line U87 and in the pediatric GBM cell line SJGBM2, suggesting that *ASAH1* confers radioresistance to GBM (Figure 3) [96,97]. Our histochemistry data utilizing patient GBM tissues revealed higher levels of *ASAH1* in irradiated tissues compared to control tissues [96]. We suggested that ASAH1 may decrease the overall GBM survival and promote recurrence, which is inevitable, by enhancing the survival of irradiated GBMs via the upregulation of *ASAH1*, leading to decreasing levels of proapoptotic ceramide molecules and increasing levels of prosurvival S1P molecules (Figure 3) [96]. Despite being resistant to radiation, these cells remained sensitive to the ASAH1 inhibitor carmofur, albeit displaying a slightly higher IC_50_ value [96]. More importantly, ASAH1 inhibitors have been proposed as radiosensitizers, on the basis of studies that illustrated a greater suppression of the growth of U87 and prostate cancer xenografts when treated with both conventional radiation therapy and ASAH1 inhibitors [87,98]. Carmofur is the only *ASAH1* inhibitor that has been used clinically to treat colorectal cancers [99,100,101]. However, carmofur has several issues that need to be addressed before it can be more widely used. It has very low solubility in aqueous solution, an intravenous formula is unavailable, and the extent to which it can penetrate the blood–brain barrier is poorly understood [91]. One strategy to improve the solubility of carmofur is to take advantage of the recently solved crystal structure of acid ceramidase to help predict how carmofur would fit in its active site and perform appropriate modifications to allow the drug to be both more soluble and potent [102]. Another strategy to combat radioresistance induced by secretion of *ASAH1* is to induce the immune system to produce autoantibodies against extracellular *ASAH1*. The benefit of developing auto-*ASAH1* antibodies was demonstrated in melanoma patients. The auto anti-*ASAH1* antibodies protected the melanoma patients from lymph node metastasis, and the loss of these antibodies could result in melanoma progression [103]. A strategy to promote the development of auto-*ASAH1* antibodies is by immunizing patients against *ASAH1*, and this may mitigate the proliferation and invasion of radioresistant GBM. Further study is needed to examine whether the auto-ASAH1 antibodies can cross the blood–brain barrier, as there is a paucity of data available regarding the benefit of auto-antibodies in treating neurological diseases. 

## 10. Identification of Novel Drug Targets to Combat Radioresistant GBM

The current standard treatment regimen for GBM includes maximal safe surgical resection, followed by radiation therapy combined with concomitant and adjuvant temozolomide [30,104]. However, recurrence of GBM—characterized by radioresistance—remains inevitable [105,106]. The absence of a major progress in the treatment of GBM maybe a result of our poor understanding of both GBM tumor biology and the mechanisms underlying the acquirement of treatment resistance in recurrent GBMs. In support of this view, very little data about the radiation effects on global gene expression at the messenger ribonucleic acid (mRNA) level in a stable radioresistant GBM model are available. Ma et al., in their transcriptome analysis of glioma within hours following irradiation, suggested that the development of radioresistance of glioma may be due to the inactivation of early proapoptotic molecules and to the late activation of antiapoptotic genes [107]. To identify radiation-responsive genes that may enable GBM cells to acquire resistance to radiation, we performed complete RNA sequencing (RNA-seq) of control tissues and our recently established stable, radioresistant U87-based GBM model [96,108]. Our study revealed that the aberrant gene expression observed in irradiated U87-10gy cells regarded, in particular, genes involved in enhancing tumor malignancy and invasion. In irradiated U87-10gy cells, we observed the upregulation of antiapoptotic genes (*BNIP3* and *SOD2*)*,* of genes promoting epithelial to mesenchymal transition, of genes with metalloendopeptidase activity, and of genes involved in the response to hypoxia (Table 1 and Table 2) [108]. Metalloproteases are known to promote tumor invasion and metastasis of many cancers by degrading the extracellular matrix [109,110]. *MME*, *MMP2*, *MMP3*, *MMP7*, *MMP12*, *ADAM9*, and *ADAM12* were shown to be upregulated in radioresistant GBMs (Table 1 and Table 2) [108]. Epithelial to mesenchymal transition, a process characterized by increased cell motility and resistance to chemo- and radiotherapy, is typically induced by *TGFB3*, which was also upregulated in irradiated U87-10gy cells [108,111,112]. Hypoxia, which is frequent in GBM, induces hypoxia-inducible factor 1-alpha (*HIF-1α*) and carbonic anhydrase 9 expressions, which in turn promote angiogenesis, migration, cell survival, proliferation, epithelial to mesenchymal transition, and radio- and chemoresistance [111,113,114]. HIF-1α and carbonic anhydrase 9 were upregulated in irradiated GBM cells [108]. 

On the other hand, we found that the downregulated genes were enriched in tumor suppressors, in genes positively regulating the immune response, in genes involved in p53-dependent apoptosis, and in cell adhesion genes. Suppressing the apoptotic potential through gene expression regulation in the irradiated cells was a proposed mechanism that explained the radioresistant nature of the irradiated GBM cells [107,108]. Many apoptotic genes discovered in our study were known to play major roles in attenuating GBM apoptosis, especially, *BBC3*, *DCC*, *BEX2*, *CASP1*, *IL1B*, and *SFRP2* [115,116,117,118,119,120]. GBM cells produce an immunosuppressive microenvironment to escape immune surveillance and enhance their own survival, and this can be accomplished through the secretion of transforming growth factor β (*TGF-β*) to block T cell activation and proliferation [121]. We identified many other downregulated genes involved in the activation of the immune system, especially genes mediating T cell antigen processing and presentation that may enable immune evasion in the radioresistant GBM cells [108]. 

Considering these global alterations of multiple biological pathways observed in irradiated GBM cells, an effective treatment targeting radioresistant GBM may require a cocktail containing multiple agents targeting multiple implicated pathways in order to have a chance to make a substantial impact on improving the overall GBM survival. 

## Figures and Tables

**Table 1:** Upregulated genes of selected enriched gene ontology categories following irradiation are shown on the basis of sets of statistically significant changes (*p* < 0.05) [108].

**GO:0006954: Inflammatory Response**		**GO:0000187: Activation of MAPK Activity**
CCL26	C–C motif chemokine ligand 26 (CCL26)	anaplastic lymphoma receptor tyrosine kinase (ALK)
CCL3	C–C motif chemokine ligand 3 (CCL3)	chondroitin sulfate proteoglycan 4 (CSPG4)
CXCL8	C–X–C motif chemokine ligand 8 (CXCL8)	dual specificity phosphatase 7 (DUSP7)
GPR68	G protein-coupled receptor 68 (GPR68)	formyl peptide receptor 1 (FPR1)
NFKBID	NFKB inhibitor delta (NFKBID)	transforming growth factor beta 3 (TGFB3)
TNFAIP3	TNF alpha-induced protein 3 (TNFAIP3)	tumor protein p73 (TP73)
TNFRSF10D	TNF receptor superfamily member 10d (TNFRSF10D)	
TNIP3	TNFAIP3 interacting protein 3 (TNIP3)	**GO:0004222: Metalloendopeptidase Activity**
XCR1	X–C motif chemokine receptor 1 (XCR1)	ADAM metallopeptidase domain 12 (ADAM12)
BDKRB1	bradykinin receptor B1 (BDKRB1)	ADAM metallopeptidase domain 19 (ADAM19)
BDKRB2	bradykinin receptor B2 (BDKRB2)	ADAM metallopeptidase with thrombospondin type 1 motif 1 (ADAMTS1)
CHST4	carbohydrate sulfotransferase 4 (CHST4)	ADAM metallopeptidase with thrombospondin type 1 motif 14 (ADAMTS14)
C3	complement C3 (C3)	bone morphogenetic protein 1 (BMP1)
FPR1	formyl peptide receptor 1 (FPR1)	matrix metallopeptidase 12 (MMP12)
GBP5	guanylate binding protein 5 (GBP5)	matrix metallopeptidase 3 (MMP3)
IL24	interleukin 24 (IL24)	matrix metallopeptidase 7 (MMP7)
IL36B	interleukin 36, beta (IL36B)	membrane metalloendopeptidase (MME)
NFATC4	nuclear factor of activated T-cells 4 (NFATC4)	teashirt zinc finger homeobox 2 (TSHZ2)
PTGER2	prostaglandin E receptor 2 (PTGER2)	
SDC1	syndecan 1 (SDC1)	**GO:0071356: Cellular Response to Tumor Necrosis Factor**
ZC3H12A	zinc finger CCCH-type-containing 12A (ZC3H12A)	C–C motif chemokine ligand 26 (CCL26)
		C–C motif chemokine ligand 3 (CCL3)
**GO:0010718: Positive Regulation of Epithelial to Mesenchymal Transition**		C–X–C motif chemokine ligand 8 (CXCL8)
BAMBI	BMP and activin membrane-bound inhibitor (BAMBI)	ankyrin repeat domain 1 (ANKRD1)
GLIPR2	GLI pathogenesis related 2 (GLIPR2)	collagen type I alpha 1 chain (COL1A1)
AXIN2	axin 2 (AXIN2)	endothelin 1 (EDN1)
COL1A1	collagen type I alpha 1 chain (COL1A1)	hyaluronan synthase 2 (HAS2)
TGFB3	transforming growth factor beta 3 (TGFB3)	periostin (POSTN)
**GO:0016477: Cell Migration**		
BAMBI	BMP and activin membrane-bound inhibitor (BAMBI)	**GO:0044344: Cellular Response to Fibroblast Growth Factor**
EPHA3	EPH receptor A3 (EPHA3)	C–X–C motif chemokine ligand 8 (CXCL8)
EPHB3	EPH receptor B3 (EPHB3)	collagen type I alpha 1 chain (COL1A1)
ERG	ERG, ETS transcription factor (ERG)	periostin (POSTN)
WWC1	WW and C2 domain containing 1 (WWC1)	snail family transcriptional repressor 2 (SNAI2)
BDKRB1	bradykinin receptor B1 (BDKRB1)	
CSPG4	chondroitin sulfate proteoglycan 4 (CSPG4)	**GO:0071560: Cellular Response to Transforming Growth Factor Beta**
COL5A1	collagen type V alpha 1 chain (COL5A1)	ankyrin repeat domain 1 (ANKRD1)
FSCN1	fascin actin-bundling protein 1 (FSCN1)	collagen type I alpha 1 chain (COL1A1)
LCP1	lymphocyte cytosolic protein 1 (LCP1)	endothelin 1 (EDN1)
PODXL	podocalyxin like (PODXL)	periostin (POSTN)
PSG2	pregnancy specific beta-1-glycoprotein 2 (PSG2)	phosphodiesterase 3A (PDE3A)
SDC1	syndecan 1 (SDC1)	
**GO:0001525: Angiogenesis**		ribonuclease A family member 1, pancreatic (RNASE1)
CXCL8	C–X–C motif chemokine ligand 8 (CXCL8)	ribonuclease A family member 2 (RNASE2)
EPHB3	EPH receptor B3 (EPHB3)	
EPHB4	EPH receptor B4 (EPHB4)	**GO:0090263: Positive Regulation of Canonical Wnt Signaling Pathway**
ACKR3	atypical chemokine receptor 3 (ACKR3)	BMP and activin membrane bound inhibitor (BAMBI)
CSPG4	chondroitin sulfate proteoglycan 4 (CSPG4)	R-spondin 3 (RSPO3)
COL8A1	collagen type VIII alpha 1 chain (COL8A1)	SRY-box 4 (SOX4)
NRXN3	neurexin 3 (NRXN3)	axin 2 (AXIN2)
NDNF	neuron-derived neurotrophic factor (NDNF)	collagen type I alpha 1 chain (COL1A1)
NRP2	neuropilin 2 (NRP2)	distal-less homeobox 5 (DLX5)
SERPINE1	serpin family E member 1 (SERPINE1)	leucine rich repeat containing G protein-coupled receptor 4 (LGR4)
ZC3H12A	zinc finger CCCH-type containing 12A (ZC3H12A)	
**GO:0008283: Cell Proliferation**		4-aminobutyrate aminotransferase (ABAT)
E2F8	E2F transcription factor 8 (E2F8)	BCL2 interacting protein 3 (BNIP3)
ERG	ERG, ETS transcription factor (ERG)	carbonic anhydrase 9 (CA9)
ROS1	ROS proto-oncogene 1, receptor tyrosine kinase (ROS1)	cytochrome P450 family 1 subfamily A member 1 (CYP1A1)
ALK	anaplastic lymphoma receptor tyrosine kinase (ALK)	egl-9 family hypoxia inducible factor 3 (EGLN3)
AXIN2	axin 2 (AXIN2)	lysyl oxidase like 2 (LOXL2)
CDC25A	cell division cycle 25A (CDC25A)	mucin 1, cell surface associated (MUC1)
CSPG4	chondroitin sulfate proteoglycan 4 (CSPG4)	periostin (POSTN)
CYP1A1	cytochrome P450 family 1 subfamily A member 1 (CYP1A1)	transforming growth factor beta 3 (TGFB3)
DLX5	distal-less homeobox 5 (DLX5)	
FSCN1	fascin actin-bundling protein 1 (FSCN1)	
FGF5	fibroblast growth factor 5 (FGF5)	
GRPR	gastrin releasing peptide receptor (GRPR)	
MYH10	myosin heavy chain 10 (MYH10)	
PDK1	pyruvate dehydrogenase kinase 1 (PDK1)	
UHRF1	ubiquitin like with PHD and ring finger domains 1 (UHRF1)	
**GO:0016049: Cell Growth**		
ROS1	ROS proto-oncogene 1, receptor tyrosine kinase (ROS1)	
EDN1	endothelin 1 (EDN1)	
IL7R	interleukin 7 receptor (IL7R)	
NDNF	neuron-derived neurotrophic factor (NDNF)	
TGFB3	transforming growth factor beta 3 (TGFB3)	

**Table 2:** Downregulated genes of selected enriched gene ontology categories following irradiation are shown on the basis of sets of statistically significant changes (*p* < 0.05) [108].

**GO:0006915: Apoptotic Process**	**GO:0008152: Metabolic Process**
BCL2 binding component 3 (BBC3)	1-acylglycerol-3-phosphate O-acyltransferase 2 (AGPAT2)
DCC netrin 1 receptor (DCC)	UDP glucuronosyltransferase family 1 member A1 (UGT1A1)
PYD and CARD domain-containing (PYCARD)	UDP glucuronosyltransferase family 1 member A10 (UGT1A10)
TNF receptor-associated factor 5 (TRAF5)	UDP glucuronosyltransferase family 1 member A3 (UGT1A3)
XIAP-associated factor 1 (XAF1)	UDP glucuronosyltransferase family 1 member A4 (UGT1A4)
Brain-expressed X-linked 2 (BEX2)	UDP glucuronosyltransferase family 1 member A5 (UGT1A5)
caspase 1 (CASP1)	UDP glucuronosyltransferase family 1 member A6 (UGT1A6)
cathepsin H (CTSH)	UDP glucuronosyltransferase family 1 member A7 (UGT1A7)
complement C5a receptor 1 (C5AR1)	UDP glucuronosyltransferase family 1 member A8 (UGT1A8)
engulfment and cell motility 1 (ELMO1)	UDP glucuronosyltransferase family 1 member A9 (UGT1A9)
interleukin 1 beta (IL1B)	acyl-CoA synthetase medium-chain family member 5 (ACSM5)
mitogen-activated protein kinase kinase 6 (MAP2K6)	acyl-CoA synthetase short-chain family member 1 (ACSS1)
nuclear receptor subfamily 4 group A member 1 (NR4A1)	acyl-CoA synthetase short-chain family member 3 (ACSS3)
phorbol-12-myristate-13-acetate-induced protein 1 (PMAIP1)	glutathione S-transferase mu 5 (GSTM5)
secreted frizzled related protein 2 (SFRP2)	haloacid dehalogenase-like hydrolase domain-containing 3 (HDHD3)
tyrosyl-tRNA synthetase (YARS)	lipase E, hormone sensitive type (LIPE)
	mannosidase alpha class 1C member 1 (MAN1C1)
**GO:0072332: Intrinsic Apoptotic Signaling Pathway by p53 Class Mediator**	
PERP, TP53 apoptosis effector (PERP)	**GO:0007155: Cell Adhesion**
PYD and CARD domain-containing (PYCARD)	CD22 molecule (CD22)
phorbol-12-myristate-13-acetate-induced protein 1 (PMAIP1)	CD9 molecule (CD9)
zinc finger matrin-type 1 (ZMAT1)	EPH receptor A4 (EPHA4)
zinc finger protein 385D (ZNF385D)	adhesion G protein-coupled receptor G1 (ADGRG1)
	amelotin (AMTN)
**GO:2000406: Positive Regulation of T Cell Migration**	basal cell adhesion molecule (Lutheran blood group) (BCAM)
PYD and CARD domain-containing (PYCARD)	brevican (BCAN)
TNF receptor superfamily member 14 (TNFRSF14)	cadherin 11 (CDH11)
integrin subunit alpha 4 (ITGA4)	collagen type IV alpha 6 chain (COL4A6)
	fasciculation and elongation protein zeta 1 (FEZ1)
**GO:0002457: T Cell Antigen Processing and Presentation**	fibulin 7 (FBLN7)
intercellular adhesion molecule 1 (ICAM1)	hemicentin 2 (HMCN2)
raftlin, lipid raft linker 1 (RFTN1)	hyaluronan synthase 1 (HAS1)
	integrin subunit alpha 11 (ITGA11)
**GO:0002282: Microglial Cell Activation Involved in Immune Response**	integrin subunit alpha 2 (ITGA2)
interleukin 33 (IL33)	integrin subunit alpha 4 (ITGA4)
toll like receptor 3 (TLR3)	integrin subunit alpha L (ITGAL)
	integrin subunit beta 8 (ITGB8)
**GO:0007165: Signal Transduction**	intercellular adhesion molecule 1 (ICAM1)
ArfGAP with RhoGAP domain, ankyrin repeat and PH domain 2 (ARAP2)	junction plakoglobin (JUP)
G kinase anchoring protein 1 (GKAP1)	laminin subunit alpha 2 (LAMA2)
G protein subunit gamma 11 (GNG11)	laminin subunit alpha 3 (LAMA3)
GULP, engulfment adaptor PTB domain-containing 1 (GULP1)	ninjurin 1 (NINJ1)
KIT proto-oncogene receptor tyrosine kinase (KIT)	protein kinase C epsilon (PRKCE)
MX dynamin like GTPase 1 (MX1)	protein kinase, X-linked (PRKX)
NDP, norrin cystine knot growth factor (NDP)	protocadherin 17 (PCDH17)
NLR family pyrin domain-containing 12 (NLRP12)	sphingosine-1-phosphate receptor 1 (S1PR1)
NLR family pyrin domain-containing 3 (NLRP3)	trophoblast glycoprotein (TPBG)
PYD and CARD domain-containing (PYCARD)	versican (VCAN)
Ras association domain family member 9 (RASSF9)	
Rho family GTPase 2 (RND2)	**GO:0045746: Negative Regulation Of Notch Signaling Pathway**
SPARC-related modular calcium binding 1 (SMOC1)	ChaC glutathione-specific gamma-glutamylcyclotransferase 1 (CHAC1)
TNF receptor-associated factor 5 (TRAF5)	MAGE family member A1 (MAGEA1)
TNF receptor superfamily member 11b (TNFRSF11B)	Hes-related family bHLH transcription factor with YRPW motif 1 (HEY1)
amyloid beta precursor protein binding family B member 1 interacting protein (APBB1IP)	maternally expressed 3 (non-protein coding) (MEG3)
androgen receptor (AR)	
basal cell adhesion molecule (Lutheran blood group) (BCAM)	**GO:0010759: Positive Regulation of Macrophage Chemotaxis**
calcitonin-related polypeptide beta (CALCB)	chemerin chemokine-like receptor 1 (CMKLR1)
caspase 1 (CASP1)	complement C5a receptor 1 (C5AR1)
chimerin 1 (CHN1)	tumor necrosis factor superfamily member 18 (TNFSF18)
complement C5a receptor 1 (C5AR1)	
fibroblast growth factor 18 (FGF18)	**GO:0006351: Transcription, DNA-Templated**
fibroblast growth factor 7 (FGF7)	CREB3 regulatory factor (CREBRF)
growth differentiation factor 15 (GDF15)	DNA damage inducible transcript 3 (DDIT3)
inositol-trisphosphate 3-kinase A (ITPKA)	E2F transcription factor 7 (E2F7)
insulin like growth factor binding protein 1 (IGFBP1)	HKR1, GLI-Kruppel zinc finger family member (HKR1)
insulin like growth factor binding protein 5 (IGFBP5)	Kruppel-like factor 2 (KLF2)
integrin subunit alpha L (ITGAL)	Kruppel-like factor 9 (KLF9)
interleukin 1 beta (IL1B)	MAGE family member A1 (MAGEA1)
interleukin 15 receptor subunit alpha (IL15RA)	MAX dimerization protein 1 (MXD1)
junction plakoglobin (JUP)	MLX interacting protein like (MLXIPL)
mitogen-activated protein kinase 10 (MAPK10)	NLR family pyrin domain-containing 3 (NLRP3)
mitogen-activated protein kinase kinase 6 (MAP2K6)	RAR related orphan receptor B (RORB)
nuclear receptor subfamily 2 group F member 1 (NR2F1)	SATB homeobox 1 (SATB1)
nuclear receptor subfamily 4 group A member 1 (NR4A1)	T-box 3 (TBX3)
nuclear receptor subfamily 4 group A member 2 (NR4A2)	TGFB-induced factor homeobox 2 like, X-linked (TGIF2LX)
phosphodiesterase 10A (PDE10A)	ZFP14 zinc finger protein (ZFP14)
phosphodiesterase 1A (PDE1A)	androgen receptor (AR)
phosphodiesterase 4D (PDE4D)	endoplasmic reticulum to nucleus signaling 1 (ERN1)
placental growth factor (PGF)	forkhead box P2 (FOXP2)
plasminogen activator, urokinase (PLAU)	hair growth associated (HR)
protein kinase AMP-activated catalytic subunit alpha 2 (PRKAA2)	hes-related family bHLH transcription factor with YRPW motif 1 (HEY1)
protein kinase C epsilon (PRKCE)	homeobox B7 (HOXB7)
protein kinase C zeta (PRKCZ)	homeobox B8 (HOXB8)
ras-related dexamethasone induced 1 (RASD1)	homeobox B9 (HOXB9)
ribosomal protein S6 kinase A2 (RPS6KA2)	interleukin 33 (IL33)
ribosomal protein S6 kinase A6 (RPS6KA6)	iroquois homeobox 5 (IRX5)
secreted and transmembrane 1 (SECTM1)	leucine zipper tumor suppressor 1 (LZTS1)
single Ig and TIR domain-containing (SIGIRR)	mitogen-activated protein kinase kinase 6 (MAP2K6)
thrombomodulin (THBD)	myelin expression factor 2 (MYEF2)
toll like receptor 3 (TLR3)	neuronal PAS domain protein 2 (NPAS2)
transducin-like enhancer of split 1 (TLE1)	nuclear factor I B (NFIB)
tumor necrosis factor superfamily member 10 (TNFSF10)	nuclear protein 1, transcriptional regulator (NUPR1)
tumor necrosis factor superfamily member 13b (TNFSF13B)	nuclear receptor coactivator 7 (NCOA7)
tumor necrosis factor superfamily member 18 (TNFSF18)	nuclear receptor subfamily 2 group F member 1 (NR2F1)
tyrosyl-tRNA synthetase (YARS)	nuclear receptor subfamily 4 group A member 1 (NR4A1)
unc-5 netrin receptor B (UNC5B)	nuclear receptor subfamily 4 group A member 2 (NR4A2)
unc-5 netrin receptor C (UNC5C)	protein kinase AMP-activated catalytic subunit alpha 2 (PRKAA2)
very low density lipoprotein receptor (VLDLR)	thyroid hormone receptor beta (THRB)
	transducin-like enhancer of split 1 (TLE1)
	tribbles pseudokinase 3 (TRIB3)
	tumor protein p63 (TP63)
	twist family bHLH transcription factor 2 (TWIST2)
	vestigial-like family member 2 (VGLL2)
	visual system homeobox 1 (VSX1)
	zinc finger and SCAN domain containing 16 (ZSCAN16)
	zinc finger family member 788 (ZNF788)
	zinc finger protein 117 (ZNF117)
	zinc finger protein 138 (ZNF138)
	zinc finger protein 20 (ZNF20)
	zinc finger protein 273 (ZNF273)
	zinc finger protein 28 (ZNF28)
	zinc finger protein 30 (ZNF30)
	zinc finger protein 320 (ZNF320)
	zinc finger protein 354B (ZNF354B)
	zinc finger protein 396 (ZNF396)
	zinc finger protein 415 (ZNF415)
	zinc finger protein 419 (ZNF419)
	zinc finger protein 433 (ZNF433)
	zinc finger protein 44 (ZNF44)
	zinc finger protein 442 (ZNF442)
	zinc finger protein 443 (ZNF443)
	zinc finger protein 468 (ZNF468)
	zinc finger protein 521 (ZNF521)
	zinc finger protein 525 (ZNF525)
	zinc finger protein 528 (ZNF528)
	zinc finger protein 549 (ZNF549)
	zinc finger protein 563 (ZNF563)
	zinc finger protein 572 (ZNF572)
	zinc finger protein 577 (ZNF577)
	zinc finger protein 625 (ZNF625)
	zinc finger protein 649 (ZNF649)
	zinc finger protein 674 (ZNF674)
	zinc finger protein 680 (ZNF680)
	zinc finger protein 71 (ZNF71)
	zinc finger protein 761 (ZNF761)
	zinc finger protein 765 (ZNF765)
	zinc finger protein 792 (ZNF792)
	zinc finger protein 799 (ZNF799)
	zinc finger protein 806 (ZNF806)
	zinc finger protein 816 (ZNF816)
	zinc finger protein 83 (ZNF83)
	zinc finger protein 845 (ZNF845)
	zinc finger protein 85 (ZNF85)
	zinc finger protein 883 (ZNF883)
	zinc finger protein 888 (ZNF888)
	zinc finger with KRAB and SCAN domains 7 (ZKSCAN7)

## Abbreviations

5-ALA	5-aminolevulinic acid
ASAH1	acid ceramidase
AML	acute myeloid leukemia
CerS	ceramide synthases
DMG	*N*-dimethyl glycine
GBM	glioblastoma
IDH	isocitrate dehydrogenase
MGMT	O6-methylguanine-methyltransferase
MRI	magnetic resonance imaging
S1P	sphingosine-1-phosphate
SPHK1	sphingosine kinase 1
SPHK2	sphingosine kinase 2
TTF	tumor-treating field
